# Development and Validation of a Novel Score for Predicting Long-Term Mortality after an Acute Ischemic Stroke

**DOI:** 10.3390/ijerph20043043

**Published:** 2023-02-09

**Authors:** Ching-Heng Lin, Ya-Wen Kuo, Yen-Chu Huang, Meng Lee, Yi-Wei Huang, Chang-Fu Kuo, Jiann-Der Lee

**Affiliations:** 1 Center for Artificial Intelligence in Medicine, Chang Gung Memorial Hospital, Taoyuan 333, Taiwan; 2 Bachelor Program in Artificial Intelligence, Chang Gung University, Taoyuan 333, Taiwan; 3 Department of Nursing, Chang Gung University of Science and Technology, Chiayi Campus, Chiayi 613, Taiwan; 4 Associate Research Fellow, Chang Gung Memorial Hospital, Chiayi 613, Taiwan; 5 Department of Neurology, Chiayi Chang Gung Memorial Hospital, Chiayi 613, Taiwan; 6 College of Medicine, Chang Gung University, Taoyuan 333, Taiwan; 7 Division of Rheumatology, Allergy, and Immunology, Chang Gung Memorial Hospital, Taoyuan 333, Taiwan

**Keywords:** acute ischemic stroke, mortality, machine learning, clinical prediction rule

## Abstract

Background: Long-term mortality prediction can guide feasible discharge care plans and coordinate appropriate rehabilitation services. We aimed to develop and validate a prediction model to identify patients at risk of mortality after acute ischemic stroke (AIS). Methods: The primary outcome was all-cause mortality, and the secondary outcome was cardiovascular death. This study included 21,463 patients with AIS. Three risk prediction models were developed and evaluated: a penalized Cox model, a random survival forest model, and a DeepSurv model. A simplified risk scoring system, called the C-HAND (history of Cancer before admission, Heart rate, Age, eNIHSS, and Dyslipidemia) score, was created based on regression coefficients in the multivariate Cox model for both study outcomes. Results: All experimental models achieved a concordance index of 0.8, with no significant difference in predicting poststroke long-term mortality. The C-HAND score exhibited reasonable discriminative ability for both study outcomes, with concordance indices of 0.775 and 0.798. Conclusions: Reliable prediction models for long-term poststroke mortality were developed using information routinely available to clinicians during hospitalization.

## 1. Introduction

In 2019, stroke was the second-leading cause of death in the world. There were 12.2 million incident cases of stroke and 6.55 million deaths from stroke. The five leading risk factors contributing to stroke death and disability combined were high systolic blood pressure, a high body mass index, high fasting plasma glucose, ambient particulate matter pollution, and smoking [1]. When a patient is admitted to the hospital with a stroke, clinicians are often required to estimate the likelihood of the patient’s mid- to long-term mortality and severe disability following the stroke event. Mortality prediction models help clinicians understand disease prognosis, and they can be used to improve communication with and the care of hospitalized patients, develop feasible discharge care plans, and provide appropriate rehabilitation services. Furthermore, the study of long-term mortality in stroke patients can provide insights into the underlying mechanisms of stroke and inform the development of new treatments and prevention strategies.

Few validated stroke prognostic models that provide predictions beyond >12 months are available [2,3,4,5]. The ASTRAL score is one of the few prognostic scoring systems validated to predict poststroke long-term mortality and is based on six items: age, stroke severity, the time delay between symptom onset or last proof of good health (in the case of an unknown onset stroke) and admission, presence of any new visual field defect, glucose at admission, and level of consciousness [6]. Although stroke severity and age are crucial predictors of outcomes after acute ischemic stroke (AIS) [5,7], many other clinical factors are associated with poststroke mortality, especially long-term mortality [8].

Machine learning (ML) and deep learning (DL) algorithms can accommodate nonlinear relationships between the risk and outcome variables when measuring the relative importance of specific risk variables [9]. These techniques leverage patterns in large datasets to predict the likelihood of an event in a given time frame. Deep learning, in particular, uses complex neural networks to model complex relationships between input variables and output prediction. The application of ML/DL algorithms results in precise and efficient prediction outcomes, making it a highly effective tool in the healthcare field, especially for providing personalized clinical care to patients with stroke, where demographic, lifestyle, and medical history data can be used to make more informed decisions about patient care and treatment plans [10]. Mortality was the most commonly predicted clinical outcome in the studies analyzed [11]. The duration of follow-up was varied, with some studies focusing on short-term mortality at time points such as 10 days [12] and 30 days [13], while others examined long-term mortality [14].

The goals of this study were to: (1) evaluate the performance of stroke prognostic models based on statistics, ML, and DL; (2) identify crucial factors for predicting long-term mortality from information routinely available to clinicians during hospitalization; and (3) develop a simplified risk scoring system using identified predicting factors and validate it for clinical practicality.

## 2. Materials and Methods

### 2.1. Study Population

**Figure 1:**
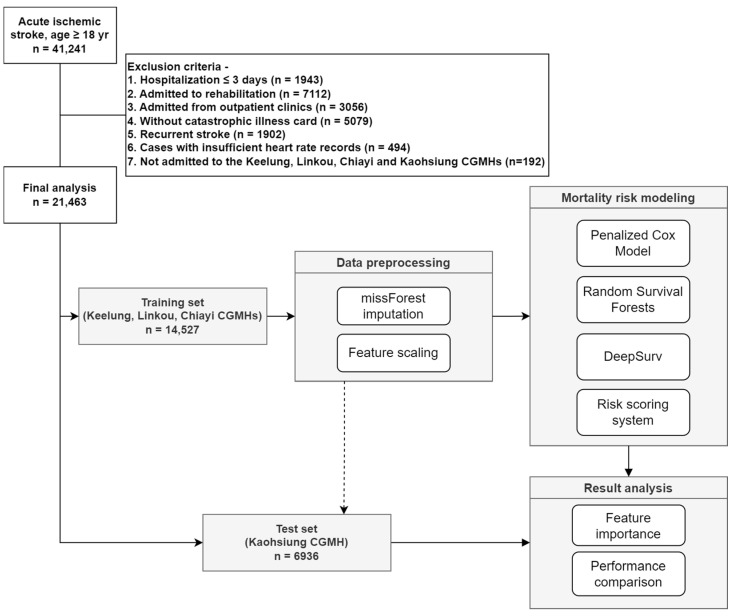
A flow chart illustrating patient selection at Chang Gung Memorial Hospital and study methodology.

This retrospective cohort study used data from the Chang Gung Research Database, the largest collection of multi-institutional electronic medical records in Taiwan [15]. All patients who had AIS (*ICD-9-CM* codes 433.01, 433.11, 433.21, 433.31, 433.81, 433.91, 434.01, 434.11, and 434.91 and *ICD-10-CM* code I63) in the first two discharge diagnosis records between January 2010 and September 2018 and who were admitted to one of the seven branch hospitals of the Chang Gung healthcare system were consecutively enrolled in this study. The initial cohort consisted of 41,241 patients with AIS, aged ≥ 18 years. The patient selection process is illustrated in Figure 1. A total of 21,463 patients with AIS were included in our final study population. The data were divided into training and test sets based on the hospital location to simulate real-world external validation (Figure 1). The study was approved by the Institutional Review Board of the Human Subject Research Program of the Chang Gung Medical Foundation (Approval No. 202100979B0C602). Waivers of informed consent were approved by the institutional review board for this retrospective study involving the secondary analysis of existing data.

### 2.2. Variables and Outcomes

Information on key demographics, laboratory results, and vital sign values was collected in this study. Stroke severity was assessed using the claims-based stroke severity index (SSI). The SSI was then converted to the National Institutes of Health Stroke Scale score using the equation: estimated National Institutes of Health Stroke Scale (eNIHSS) = 1.1722 × SSI − 0.7533 [16]. Data on systolic blood pressure, diastolic blood pressure, heart rate (HR), respiratory rate, and pulse pressure in the first three days of hospitalization were collected (see the detailed data processing description in the Supplementary Materials).

Regarding the outcomes of this study, the primary outcome was all-cause mortality, and the secondary outcome was cardiovascular death. Cardiovascular diseases were identified using the *ICD-10-CM* codes I00–I99. The data on patients with AIS in this study were linked to the National Registry of Deaths provided by the Ministry of Health and Welfare in Taiwan for 2010–2018. The National Registry of Death holds information on the primary and contributing causes of death and the date of death for all citizens.

### 2.3. Data Preprocessing and Modeling

Figure 1 illustrates the study flow diagram. The data were divided into training and test sets. A random forest model [17] trained on the training set was used to impute the missing values in the training and test sets. The missing data rates for the training and test sets were 6% and 4%, respectively. We fitted the standard scaler to standardize features by removing the mean and scaling to unit variance on the training set, and then used the scaler to transform the data on the training and test sets. Three risk assessment models were developed in this study: a penalized Cox model (L1 regularization) based on statistics, a random survival forest (RSF) model [18] based on ML, and a DeepSurv [19] model based on DL. In addition, a simplified risk scoring system was created based on the regression coefficients of the multivariate Cox model for both study outcomes [20]. The variables adopted in the scoring system were selected from the top five crucial variables in the penalized Cox model. Because age was found to be significantly associated with the risk of poststroke mortality, the β coefficient of age (per 10 years) was used as the number of regression units that represented one point in the final scoring system. The points assigned to other risk factors were obtained by dividing each β coefficient by that of age and rounding the score to the nearest integer [21]. If the nearest integer was zero, this risk factor would be removed from the final scoring system. Although ML/DL approaches can report feature importance in some ways, to our best knowledge, there is no appropriate way to transform feature importance into a simple scoring system. The limitations include the lack of transparency in these black-box ML/DL approaches and the complex nonlinear operations in the feature generation process.

### 2.4. Statistical Analysis

We used the test set to evaluate the predictive performance of the model. Model performance was evaluated using the concordance index (C-index). The chi-square test was used for categorical variables and an unpaired Student’s t test for continuous variables to compare population characteristics. A *p*-value of <0.05 was considered statistically significant. Feature importance was computed from permutation importance [22] for the RSF model and coefficient values for the penalized Cox model. Model development and all analyses were performed using scikit-survival (version 0.17.1), pycox (version 0.2.3), and R (version 4.0).

## 3. Results

### 3.1. Patient Characteristics

A total of 21,463 adult patients with AIS were enrolled in this study (age, 67.33 ± 12.93 years; 38.15% female patients). The median follow-up duration was 3.2 years (interquartile range of 1.4–5.6 years). The training set consisted of the data of 14,527 patients from the Keelung, Linkou, and Chiayi branch hospitals. The test set consisted of the data of 6936 patients from the Kaohsiung branch hospital (Table 1).

### 3.2. Variable Importance Analysis

**Figure 2:**
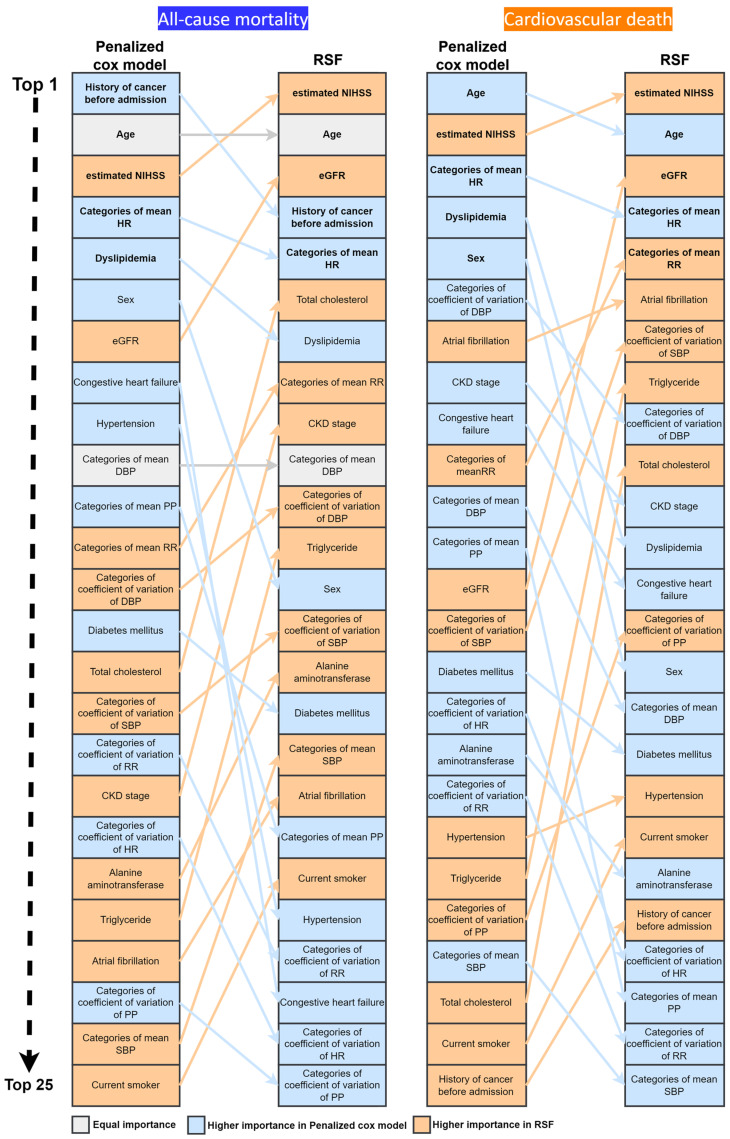
List of variables ranked by their importance to all-cause mortality and cardiovascular death in the penalized Cox model and RSF model. Abbreviations: RSF, random survival forest; NIHSS, National Institute of Health Stroke Scale; eGFR, estimated glomerular filtration rate; HR, heart rate; SBP, systolic blood pressure; DBP, diastolic blood pressure; RR, respiration rate; PP, pulse pressure; and CKD, chronic kidney disease.

Figure 2 presents the ranking of importance of the selected variables for all-cause mortality and cardiovascular death. Although the RSF model uses a nonlinear feature selection method and the penalized Cox model uses a linear feature selection method, the top five crucial variables for the risk prediction of all-cause mortality were similar in the RSF and penalized Cox models, except that dyslipidemia was the fifth crucial predictor in the penalized Cox model and eGFR was the third vital predictor in the RSF model. For the risk prediction of cardiovascular death, the history of cancer before admission was not among the top five crucial variables. In addition to age, stroke severity, and mean HR, dyslipidemia and sex were selected in the penalized Cox model, and eGFR and the mean respiratory rate were selected in the RSF model as among the top five crucial predictors for cardiovascular death.

### 3.3. Model Performance Evaluation

**Figure 3:**
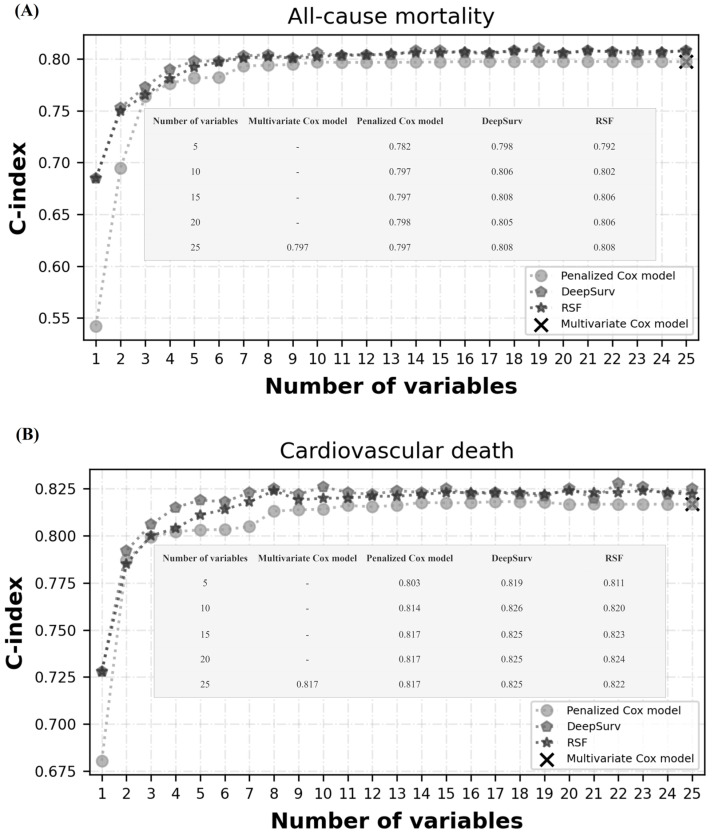
Performance of models with a varying number of variables. (**A**) Models’ performance in all-cause mortality prediction. (**B**) Models’ performance in cardiovascular death prediction.

We evaluated the performance of the three models by using the test set. The performance of the multivariate Cox model was considered the baseline reference. Figure 3 presents the performance of the models with a varying number of variables. The RSF model was evaluated with variables ranked by permutation importance. The penalized Cox model was evaluated with variables ranked by coefficient values. For the prediction of all-cause mortality, the DeepSurv model achieved the highest performance when the top 15 crucial variables (C-index = 0.808) were adopted. Moreover, the DeepSurv model achieved the highest performance when the top 10 crucial variables were used to predict cardiovascular death (C-index = 0.826). There was no significant difference in model performance if all variables were included in the analysis.

### 3.4. Risk Scoring Systems for All-Cause Mortality and Cardiovascular Death

We developed a simplified risk scoring system for clinical practicality. We used the top five crucial variables from the penalized Cox model to develop the risk scoring system for all-cause mortality and cardiovascular death. From the evaluation results of model performance, the performance of all the models approached a C-index of 0.8 when the top five crucial variables were included in the analysis.

The final risk score was named the C-HAND score (history of Cancer before admission, Heart rate, Age, eNIHSS, and Dyslipidemia). As presented in Table 2, the risk score for all-cause mortality consisted of four positive predictors and one negative predictor, with 2 points for a history of cancer before admission, 1 point for every 10 years of age, 1 point for moderate stroke severity (NIHSS 6–13), 2 points for severe stroke severity (NIHSS > 13), 1 point for a mean HR of 70–90 beats per minute, 2 points for a mean HR > 90 beats per minute, and minus 1 point for dyslipidemia. The risk score for cardiovascular death consisted of three positive predictors and one negative predictor, with 1 point for every 10 years of age, 1 point for moderate stroke severity (NIHSS 6–13), 3 points for severe stroke severity (NIHSS > 13), 1 point for a mean HR of 70–90 beats per minute, 2 points for a mean HR > 90 beats per minute, and minus 1 point for dyslipidemia.

**Figure 4:**
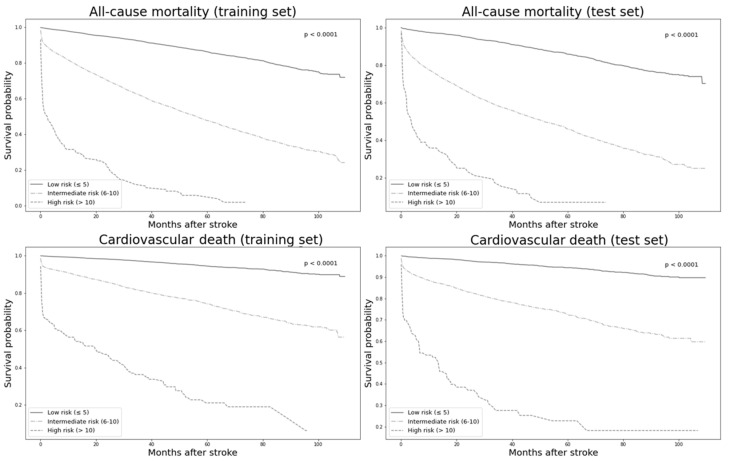
Kaplan–Meier curves for low (C-HAND score ≤ 5), intermediate (5 < C-HAND score ≤ 10), and high (C-HAND score > 10) risk groups for all-cause mortality and cardiovascular death in training and test sets.

The incidence rates of all-cause mortality and cardiovascular death in the training cohort, test cohort, and entire study population are provided in Supplementary Figure S1. The overall incidence rates of all-cause mortality and cardiovascular death were 80.4/1000 and 33.7/1000 person-years, respectively. The median total risk scores for all-cause mortality and cardiovascular death were 5 points. We stratified the total risk scores into three risk categories: low (≤5), intermediate (5–10), and high (>10). Approximately 1.1% and 2.4% of patients scored >10 points for all-cause mortality and cardiovascular death, respectively, yielding the incidence rates of 973.5/1000 person-years for all-cause mortality and 492.4/1000 person-years for cardiovascular death. By contrast, among patients with a score of ≤5 points, the incidence rates of all-cause mortality and cardiovascular death were only 31.6/1000 and 11.7/1000 person-years, respectively. Figure 4 presents Kaplan–Meier curves for both all-cause mortality and cardiovascular death in the training and test cohorts. The log-rank test revealed significant differences in survival curves (*p* < 0.001). The C-indices of the risk scoring system for all-cause mortality and cardiovascular death were 0.778 and 0.812, respectively, for the training set. With C-indices of 0.775 and 0.798, respectively, the scoring system for all-cause mortality and cardiovascular death demonstrated reasonable discriminative ability for the test set.

## 4. Discussion

We developed and validated three poststroke prognostic models (the penalized Cox model based on statistics, the RSF model based on ML, and the DeepSurv model based on DL) with routinely available clinical variables during hospitalization. According to the verification of prediction models based on various statistics, ML, and DL algorithms, all the models achieved high and consistent performance for predicting poststroke long-term mortality. No significant difference was observed in the predictive performance of the three models. This result implies that a reliable prediction model can be built using existing data in our database.

A simplified risk scoring system, the C-HAND score, was developed and externally validated; it can aid clinicians in determining the risks of all-cause mortality and cardiovascular death after AIS. The results revealed that the score can reliably predict long-term poststroke mortality, thus extending its applicability. The combination of speed (quick calculation after 3 days of hospitalization), simplicity (four to five variables), and reliability for predicting long-term mortality highlights that the C-HAND scoring system is a valuable clinical and research tool.

The Northern Manhattan Stroke Study revealed that a large proportion of long-term deaths after ischemic stroke are nonvascular in origin [23]. Although cancer is a crucial cause of all-cause mortality after stroke, not all currently available prediction models include the history of cancer as a predictor of long-term all-cause mortality [2,6,7,24,25]. In the current study (Figure 2), the history of cancer before admission was a major predictor of all-cause mortality in both the penalized Cox model and the RSF model. By contrast, a history of cancer before admission was not a crucial predictor of cardiovascular death in this study. Therefore, the history of cancer before admission was included as an important predictor of all-cause mortality in the C-HAND score.

In the penalized Cox model, with the exception of a history of cancer before admission, the top five crucial predictors were the same for all-cause mortality and cardiovascular death (Figure 2). Because cardiovascular diseases and cancer are the two leading causes of death after stroke, most predictors overlap, as expected, in the prediction models for cardiovascular death and all-cause mortality. The importance of cancer history becomes more apparent when discussing the risk of long-term rather than short-term all-cause mortality because cancer has been reported to be the leading cause of death in ischemic stroke after the first year [26].

Some vascular risk factors and vascular comorbidities were included in the PLAN score [2], the THRIVE score [4], and the iScore [24]. However, these factors were not included in the ASTRAL [7] or C-HAND scores. According to the improvement in treatment concept, most clinicians are more actively managing vascular risk factors than before. Therefore, recurrent stroke and vascular event rates have declined substantially [27]. In the current study, traditional vascular risk factors were not among the top five crucial predictors of all-cause mortality or cardiovascular death in the penalized Cox model. By contrast, dyslipidemia was inversely associated with long-term mortality in this study and a previous study [7]. Although dyslipidemia is a well-known risk factor for cardiovascular disease, it is not a risk factor for post-stroke mortality. In several large cohorts, blood lipid levels and body mass index have generally been inversely associated with poststroke mortality [28,29]. These results imply that nutritional status, as reflected by lipid concentrations, may be more associated with long-term mortality than the vascular risk of dyslipidemia.

Although significant advances have been made in prevention strategies, the role of HR control in the prevention of cardiovascular disease and the improvement of poststroke survival is still inconclusive and is an overlooked research topic. HR is a crucial predictor of all-cause mortality or cardiovascular death in the C-HAND score. In contrast to most of the nonmodifiable predictors in the scoring system, HR is a relatively modifiable factor. A clinical trial should be conducted to confirm the hypothesis that using appropriate medications to reduce HR can improve long-term survival after AIS.

This study has the following strengths: a large sample size of the validation cohort, long-term follow-up, and definite endpoints. We used several analytical methodologies (statistics, ML, and DL) to confirm the study results. However, the present study has several limitations. First, this study only included the first-ever ischemic stroke during the study period. Recurrent ischemic stroke after the index stroke was excluded in this study. Second, patients hospitalized for less than 3 days were excluded from this study. As a result, some patients with mild stroke may have been overlooked and missed in this study. Third, our population included patients admitted to medical centers or metropolitan hospitals in Taiwan, and the findings may not apply to patients in other settings.

## 5. Conclusions

In conclusion, C-HAND is a simple score that reliably predicts long-term mortality risk in patients with AIS. The scoring system identified HR as a novel modifiable factor. Additional prospective studies should investigate the effect of HR-reducing therapies on poststroke mortality.

## Figures and Tables

**Table 1:** Demographic and baseline characteristics of the overall, training, and test study populations.

	Training Set N = 14,527	Test Set N = 6936	*p*-Value
Age (year)			0.997
Mean (SD)	67.33 (13.11)	67.33 (12.55)	
Male	8914 (61.4)	4360 (62.9)	0.036
eNIHSS			<0.001
Mean (SD)	6.97 (5.59)	7.83 (6.40)	
Hypertension	10,918 (75.2)	5090 (73.4)	0.006
Diabetes mellitus	5959 (41.0)	2633 (38.0)	<0.001
Dyslipidemia	6390 (44.0)	3278 (47.3)	<0.001
Atrial fibrillation	2129 (14.7)	955 (13.8)	0.087
Coronary artery disease	1236 (8.5)	673 (9.7)	0.004
Congestive heart failure	763 (5.3)	410 (5.9)	0.051
History of cancer before admission	926 (6.4)	510 (7.4)	0.008
Current smoker	4246 (29.2)	1672 (24.1)	<0.001
Total cholesterol (mmol/L)			0.074
Mean (SD)	6.69 (22.86)	7.34 (26.16)	
Triglyceride (mmol/L)			0.085
Mean (SD)	2.47 (10.20)	2.76 (11.95)	
eGFR (mL/min/1.73 m^2^)			<0.001
Mean (SD)	1.25 (1.28)	1.38 (1.66)	
Alanine transaminase (U/L)			0.222
Mean (SD)	26.63 (25.41)	27.36 (46.21)	
Mean heart rate (beats per minute)			<0.001
Mean (SD)	74.31 (11.59)	75.89 (10.46)	
Mean systolic blood pressure (mmHg)			<0.001
Mean (SD)	152.20 (20.11)	148.47 (18.35)	
Mean diastolic blood pressure (mmHg)			<0.001
Mean (SD)	86.09 (11.39)	82.09 (10.17)	
Mean pulse pressure (mmHg)			0.094
Mean (SD)	65.93 (14.98)	66.29 (13.68)	
Mean respiratory rate (breaths per minute)			<0.001
Mean (SD)	17.75 (1.35)	17.54 (1.40)	
CV of heart rate			<0.001
Mean (SD)	0.10 (0.04)	0.10 (0.04)	
CV of systolic blood pressure			<0.001
Mean (SD)	0.11 (0.03)	0.10 (0.03)	
CV of diastolic blood pressure			<0.001
Mean (SD)	0.11 (0.04)	0.11 (0.04)	
CV of pulse pressure			<0.001
Mean (SD)	0.19 (0.06)	0.19 (0.06)	
CV of respiratory rate			<0.001
Mean (SD)	0.08 (0.04)	0.09 (0.04)	

Quantitative variables are summarized as the mean (standard deviation), and categorical variables are presented as a number (percentage). The unpaired *t* test was used for comparisons of continuous variables, and Pearson’s chi-squared test was used for comparisons of categorical variables between the training set and the test set. Abbreviations: SD, standard deviation; Q, quartile; eNIHSS, estimated National Institute of Health Stroke Scale; eGFR, estimated glomerular filtration rate; and CV, coefficient of variation.

**Table 2:** Cox model and risk scoring system for long-term mortality.

	HR	95% CI	β Coefficient	*p-*Value	Points
All-Cause Mortality					
History of cancer before admission	2.32	2.07–2.60	0.84	<0.0001	2
Age (per 10 years)	1.63	1.05–1.05	0.49	<0.0001	1
Stroke severity					
Moderate (eNIHSS 6–13)	1.46	1.33–1.60	0.38	<0.0001	1
Severe (eNIHSS > 13)	3.29	3.01–3.56	1.19	<0.0001	2
Mean heart rate (beats per minute)					
≥60 and <70	1.14	0.98–1.31	0.13	0.0883	0
≥70 and <80	1.58	1.37–1.81	0.45	<0.0001	1
≥80 and <90	2.01	1.73–2.33	0.70	<0.0001	1
≥90	2.88	2.46–3.37	1.06	<0.0001	2
Dyslipidemia	0.71	0.66–0.76	−0.35	<0.0001	−1
Cardiovascular Death					
Age (per 10 years)	1.05	1.05–1.06	0.52	<0.0001	1
Stroke severity					
Moderate (eNIHSS 6–13)	1.66	1.43–1.93	0.57	<0.0001	1
Severe (eNIHSS > 13)	5.57	4.94–6.27	1.72	<0.0001	3
Mean heart rate (beats per minute)					
≥60 and <70	1.13	0.89–1.43	0.12	0.322	0
≥70 and <80	1.43	1.13–1.80	0.36	0.003	1
≥80 and <90	1.88	1.48–2.39	0.63	<0.0001	1
≥90	2.53	1.97–3.24	0.93	<0.0001	2
Dyslipidemia	0.71	0.64–0.80	−0.33	<0.0001	−1
Male sex	1.24	1.12–1.38	0.21	<0.0001	0

Abbreviations: HR, hazard ratio; CI, confidence interval; and eNIHSS, estimated National Institutes of Health Stroke Scale.

## Data Availability

The data that support the findings of this study are available from Chang Gung Memorial Hospital, but restrictions may apply to the availability of these data, which were approved by the individual hospital IRB for the current study and are not publicly available. However, processed datasets can be requested and made available from the authors with the permission of Chang Gung Memorial.

## Supplementary Materials

The following supporting information can be downloaded at: https://www.mdpi.com/article/10.3390/ijerph20043043/s1, Detailed data processing; Figure S1: Incident rate of all-cause mortality and cardiovascular death across risk scores.
