# Supplementary material for: Development and Validation of a Novel Score for Predicting Long-Term Mortality after an Acute Ischemic Stroke
Source: Int J Environ Res Public Health. 2023 Feb 9;20(4):3043. doi: 10.3390/ijerph20043043 (PMC9961287; doi:10.3390/ijerph20043043)
Supplement: Supplementary file 1 [file ijerph-20-03043-s001.zip › ijerph-2125306-supplementary.pdf]

Detailed data processing

For further analysis, the mean and coefficient of the variation of HR, systolic blood pressure, diastolic blood pressure, pulse pressure, and respiratory rate were derived from the recorded vital sign values and subdivided into five subgroups (Supplementary Table 1). The estimated glomerular filtration rate (eGFR) was determined using the Modification of Diet in Renal Disease equation as follows: eGFR (mL/min/1.73 m^2) $=186 \times (\text{serum creatinine})^{-1.154} \times (\text{age})^{-0.203} \times 0.742$ (if female). Chronic kidney disease was classified into five stages: stage 1 (eGFR $\geq$ 90), stage 2 (eGFR = 60–89), stage 3 (eGFR = 30–59), stage 4 (eGFR = 15–29), and stage 5 (eGFR < 15) (all eGFR in mL/min/1.73 m^2). Stroke severity was assessed using the claims-based stroke severity index (SSI). The SSI was converted into the National Institutes of Health Stroke Scale score by using the following equation: estimated National Institutes of Health Stroke Scale (eNIHSS) = $1.1722 \times \text{SSI} - 0.7533$. Stroke severity was categorized into mild (eNIHSS score $\leq$ 5), moderate (eNIHSS score of 6–13), and severe (eNIHSS score >13).

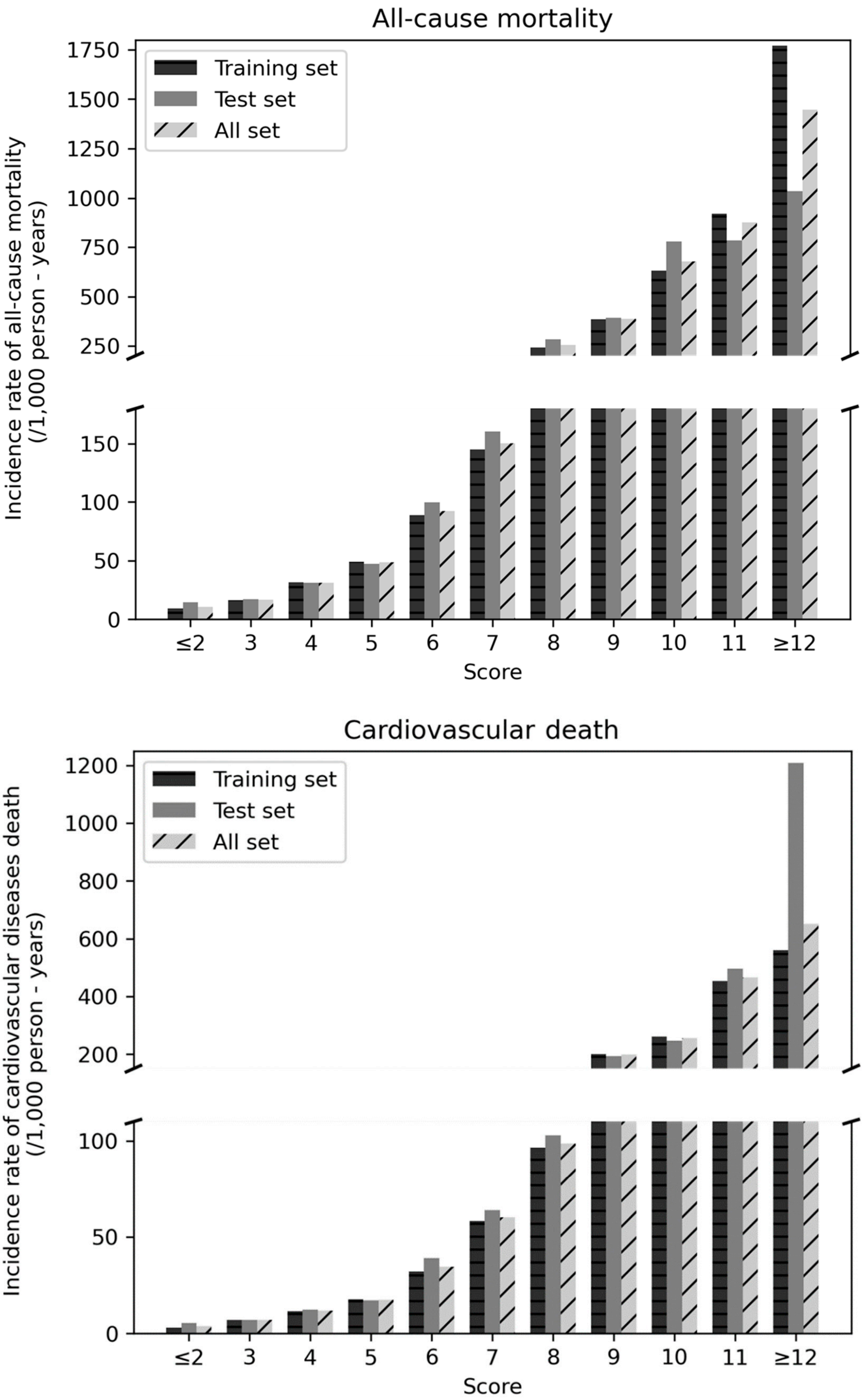

Figure S1. Incident rate of all-cause mortality and cardiovascular death across risk scores
